# Building a biomedical pipeline: the impact of the Idaho IDeA INBRE summer research experience at a primarily undergraduate institution

**DOI:** 10.1152/advan.00232.2020

**Published:** 2021-06-01

**Authors:** Sara J. Heggland, Carolyn J. Hovde, Scott A. Minnich, Linda E. Liou, Richard L. Daniels

**Affiliations:** ^1^Department of Biology, The College of Idaho, Caldwell, Idaho; ^2^Department of Animal, Veterinary, and Food Science, University of Idaho, Moscow, Idaho; ^3^Idaho INBRE Program, University of Idaho, Moscow, Idaho

**Keywords:** biomedical, Idaho, INBRE, undergraduate, undergraduate research

## Abstract

Idaho Institutional Development Award (IDeA) Network for Biomedical Research Excellence (INBRE) aims to build biomedical research capacity and enhance the scientific and technology knowledge of the Idaho workforce. A key INBRE Program at The College of Idaho, a primarily undergraduate institution of 1,100 students, is a 10-wk summer fellows research experience. This report documents outcomes from 2005 to present, including demographic trends, faculty and student research productivity, self-reported gains, educational attainment, and career outcomes. Of 103 participants, 83.7% were from Idaho, 26.7% from rural areas, and 23.9% first-generation college students. Faculty and student research productivity (conference presentations and peer-reviewed publications) increased threefold. We found that 91.4% of fellows entered a scientific- or healthcare-related career and that 70.7% completed or are currently enrolled in postgraduate training (51.7% doctoral and 19.0% master’s level). Anonymous surveys were uniformly positive, with gains in self-confidence and independent laboratory work. Open-ended responses indicated students valued mentoring efforts and improved awareness of scientific opportunities and competitive preparation for postgraduate training. Lastly, we observed that student research involvement increased college-wide during the award period. These data suggest that the summer fellows program is successfully meeting National Institutes of Health IDeA goals and serving as a pipeline to future health research careers and a scientifically trained Idaho workforce.

## INTRODUCTION

The Institutional Development Award (IDeA) Network of Biomedical Excellence (INBRE), administered by the National Institute of General Medicine Sciences at the National Institutes of Health (NIH), aims to build biomedical and behavioral research capacity in states that historically have had low levels of NIH funding. Idaho is one of 23 states plus Puerto Rico that is eligible for the IDeA Program awards and has participated in INBRE since 2001 (BRIN from 2001 to 2004). INBRE serves to cultivate the development, coordination, and sharing of research resources and expertise resulting in the expansion of biomedical research capacity and the number of competitive investigators in IDeA-eligible states. Each state funded by INBRE is tasked with building a statewide network centered on a biomedical research theme. The overarching goals of each statewide INBRE network is to foster scientific discovery, collaboration, and productivity among institutions of higher learning including research-intensive universities, primarily undergraduate institutions (PUIs), community colleges, and minority serving institutions thereby creating a pipeline for students to continue in health research careers and enhancing the science and technology knowledge of the state’s workforce.

**Figure 1.**
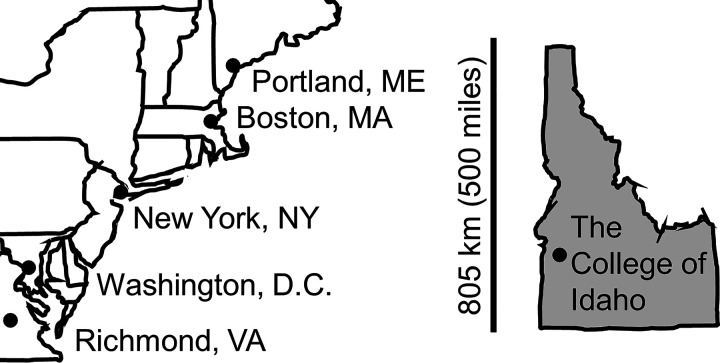
To-scale map of Idaho next to states and main cities of the eastern United States. The bar shows distance from north to south. The College of Idaho is in southwestern Idaho.

Idaho faces unique challenges in geography, education, healthcare, and research. The state of Idaho is geographically large and remote. The landscape is rugged with vast expanses of mountainous terrain and wilderness areas, with no major highway that links the southern and northern parts of the state. Figure 1 shows a to-scale map comparing Idaho to several states on the U.S. East Coast for reference. Even though Idaho is the 11th largest state by size, it is one the least populous at 1.7 million people (1). Idaho’s population density of 21.6 people/sq. mile is far less than Maine (43.6 people/sq. mi), Virginia (216 people/sq. mile), and New Jersey (1,208 people/sq. mile) (2). Consequently, much of Idaho (including 35 of 44 counties), is classified as rural by the U.S. Health Resources & Services Administration and other federal agencies. The high school graduation rate ranks Idaho at 43rd in the nation (3). Of Idaho high school graduates who enter a 4-yr public institution, only 50.7% leave with a degree, with only three states ranking lower than Idaho (4). Investment in public education continues to lag behind other states, with Idaho ranking last among states for spending per pupil (5). Like education, health disparities are pronounced in Idaho. For example, Idaho ranks 50th in the nation for the number of primary care physicians/capita (6). Similarly, biomedical research in the state is underfunded, with Idaho ranking 50th in research funding per capita by the NIH, calculated using current state population figures. One historical reason for this is the lack of a medical school in Idaho. To compensate, Idaho has partnered with the University of Washington since the 1970s to train physicians to practice in rural areas. This partnership in the WWAMI program (Washington, Wyoming, Alaska, Montana, and Idaho) is a highly recognized rural medicine program for western states lacking medical schools and currently enrolls 40 Idaho students per year. Though a new osteopathic medical school, established in Idaho in 2016, may address some of the healthcare challenges, these statistics point to the continued need for investments in higher education and improvements in access to biomedical-oriented careers in healthcare and research.

**Figure 2.**
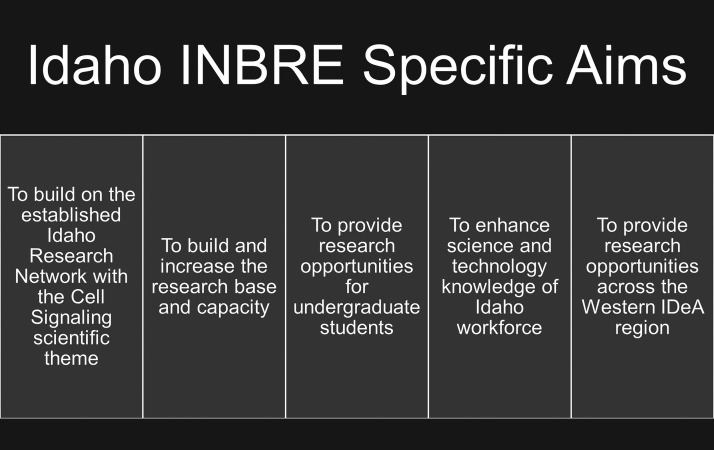
Specific aims of the statewide Idaho IDeA INBRE Program. These aims have been broadly similar across multiple funding renewals from 2005 to present. The summer research fellowship program at The College of Idaho works toward these goals. IDeA, Institutional Development Award; INBRE, IDeA Network of Biomedical Excellence.

Since 2001, the Idaho IDeA INBRE Program has created a network of 11 institutions to address Idaho’s geographical, educational, and healthcare-related challenges by supporting biomedical research and career pipelines across the state. The aims of the Idaho INBRE have remained broadly consistent throughout the award and its four renewals (Fig. 2). In support of these aims, the Idaho INBRE network has greatly improved collaborations, networks, and shared resources to advance biomedical research capacity (https://inbre.uidaho.edu/). Some of the most profound outcomes of these efforts have occurred on the campuses of Idaho’s PUIs, including The College of Idaho. The College of Idaho is Idaho’s only private, undergraduate liberal arts college and is located in Southwest Idaho (Fig. 1). With an enrollment of ∼1,100 students. The College of Idaho attracts a diverse and academically well-prepared student body. Though the majority of students are from Idaho and the Pacific Northwest (68%), international students represent 88 different countries and make up a significant portion of the population (18%). Nearly 30% of students are Hispanic, African-American, Asian-American, American Indian, or multiethnic. Additionally, 51% of the students are women, and 33% are first generation scholars (neither parent attended college). The student to faculty ratio is 11:1 with 81% of full-time faculty holding a Ph.D. or terminal degrees in their field. The biology and chemistry departments are comprised of seven and four tenure-track faculty, respectively. On average, the biology and chemistry departments graduate ∼51 undergraduate students per year in biology- and chemistry-related majors.

**Figure 3.**
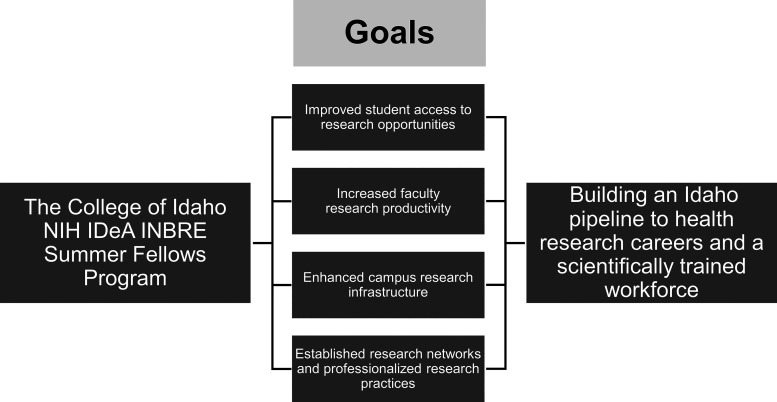
Goals of The College of Idaho INBRE summer fellows program. Participation in the summer fellows program led to 4 goals. In turn, these goals act to form a pipeline for students to enter health research careers and the scientifically trained workforce. IDeA, Institutional Development Award; INBRE, IDeA Network of Biomedical Excellence.

Since 2005, The College of Idaho has experienced a transformative paradigm shift in the culture of scientific research and research training capacity. This transformation is directly tied to participation in the Idaho INBRE Program. In pursuit of the five aims outlined in Fig. 2, Idaho INBRE has provided resources for research laboratory renovations, purchase of new instrumentation, support for core facility usage at other institutions, networking and collaboration opportunities, bioinformatics seminars, faculty development, and integration of bioinformatics into the curriculum. However, the largest INBRE-supported program at The College of Idaho has been student research opportunities during the academic year and summer with faculty mentors. In particular, the INBRE summer fellows program has been the focal point of INBRE support on The College of Idaho campus. This full-time,10-wk immersive research experience is undertaken with the supervision of a faculty mentor on projects that broadly aligned with the Idaho INBRE program-specific scientific theme of “cell signaling.” Students choose to work with a faculty mentor in chemistry or biology studying such diverse topics as sensory biology, skeletal toxicology, cellular locomotion, environmental toxicology, plant-derived chemical compounds with therapeutic potential, and small molecule inhibitors to help combat potentially pathogenic microorganisms. The summer program culminates each year with a statewide INBRE Research Conference that showcases IDeA-funded research from faculty, postdoctural researchers, graduates, and undergraduates. The goals of the program are *1*) improve student access to research opportunities; *2*) increase faculty research productivity; *3*) enhance campus research infrastructure; and *4*) establish research networks and professionalized research practices (Fig. 3).

The goals of The College of Idaho summer fellows program are consistent with other summer research experiences for undergraduates that are now a mainstay in most college science programs and are a universal component of NIH IDeA INBRE programs. Intensive, full-immersion research experiences are shown to improve student interest in science and increases their ability to self-identity as a scientist. Furthermore, these experiences cultivate many skills essential for (though not limited to) science careers, such as writing and oral communication, self-confidence, and persistence (7–9). Another key component of the summer research experience is the faculty mentor. The student’s relationship with their faculty mentor is integral in helping shape and identify their career path (10, 11). However, providing access to these programs is often challenging and is limited by the funding available to support undergraduate work in an active research laboratory. Thus, multi-institutional efforts such as the Idaho INBRE Program collectively work to provide these critical biomedical research and training opportunities to institutions across the state, including PUIs such as The College of Idaho.

Here we present an analysis of The College of Idaho summer INBRE fellows program since its inception in 2005. Specifically, we examine demographic trends of students that have participated as summer fellows, research productivity of students and faculty, and the educational attainment and career outcomes of former fellows. Furthermore, we solicited anonymous feedback from the former summer fellows regarding their experience. Our findings demonstrate that our program is successful in providing students with biomedical research training and serves as pipeline to health research careers and a scientifically trained Idaho workforce. We also report the impact of the INBRE program on increasing faculty research productivity, enhancing campus research infrastructure, and establishing research networks and professionalized research practices.

## METHODS

### The College of Idaho INBRE Summer Fellows Program

The program is a 10-wk student biomedical research experience with a College of Idaho faculty mentor in the biology or chemistry department. Over time, a number of other elements have been incorporated into the summer program, including formalized safety and responsible conduct of research (RCR) training, oral communication workshop, and poster practice sessions. In 2019 the summer program expanded to include all student and faculty science researchers (INBRE and non-INBRE funded) with weekly programmatic activities centered on professional development, career planning, networking, and research skill development. The experience culminates with a poster presentation that highlights each student's research at the annual Idaho statewide INBRE summer research conference. Since 2005, 103 students from The College of Idaho have completed the INBRE summer fellows program.

The selection process is rigorous and competitive. A student submits an application packet to the Idaho INBRE Administrative Core Office at the University of Idaho that includes grade point average, demographic information, first-generation college student status (whether either parent completed at least a bachelor's degree), college transcript, major field of study, expected graduation date, two letters of recommendation, short essays on prior research experience, career goals, what the student hopes to gain from the summer INBRE research experience, and an obstacles the student overcame to obtain a college education. Students indicate preference for their faculty mentor at the following participating institutions in Idaho: Boise State University, Boise VA Medical Center, Idaho State University, Lewis-Clark College, Northwest Nazarene University, The College of Idaho, and University of Idaho. Applications from students who select a College of Idaho faculty mentor are reviewed by INBRE-funded faculty at The College of Idaho. Final selection of The College of Idaho INBRE fellows is made by each faculty mentor based on the following criteria set by the Idaho INBRE program. These criteria are considered as a “whole” and not necessarily in the order listed: *1*) interest in biomedical science and research; *2*) academic record; *3*) letters of recommendation; *4*) date of expected graduation; *5*) family educational background; *6*) state in which student graduate from high school with preference is given to those graduating from an Idaho high school or high school in a Western INBRE state; and *7*) obstacles student overcame to obtain an education.

### Data Collection and Compilation

Data were collected from a number of sources. The Idaho INBRE Administrative Core created and employs an INBRE Reporting Database to facilitate activity tracking and evaluation of annual reporting by all Idaho INBRE funded institutions. This database was mined for all The College of Idaho INBRE fellows from 2005 to 2019 for information on gender, first-generation college student status, and whether or not the student had most recently attended a high school classified as rural. High schools were classified using the U.S. Health Resources & Services Administration’s web-based tool: Rural Health Grants Eligibility Analyzer (https://data.hrsa.gov/tools/rural-health). The Idaho INBRE Administrative Core also provided information on conference presentations and publications by The College of Idaho INBRE-funded faculty and students. The College of Idaho Registrar’s office provided information to calculate graduation rate of INBRE fellows. Institutional demographic data were provided by The College of Idaho Associate Vice President of Institutional Effectiveness. Faculty provided information on non-INBRE funded student researchers in order to determine total number of student summer researchers in biology and chemistry. These were students supported internally by the college or by external federal or nonfederal awards other than INBRE. For data regarding career outcomes and postgraduate programs, we omitted students who are currently enrolled at The College of Idaho.

### Postsurvey of Former College of Idaho INBRE Summer Fellows

Email addresses for the 103 summer research fellows were collected from faculty who kept in contact with former fellows, social media messaging, and The College of Idaho Alumni Office. An online survey was created using Microsoft Forms and the link was emailed to each former INBRE summer fellow on June 25, 2020 to be completed by July 7, 2020. With informed consent, the survey asked for demographic information, Likert scale questions on their experience as an INBRE fellow at The College of Idaho, highest degree earned, career choice, postgraduate education, and open-ended questions on what was the most impactful part(s) of being involved in INBRE-funded research at The College of Idaho. The survey was approved by The College of Idaho Institutional Review Board (IRB Federal Registration IORG0005643).

### Statistical Analysis

Data on conference presentations and publications were calculated as an average per 5 yr (2005–2009, 2010–2014, and 2015–2019) and analyzed using a one-way analysis of variance followed by a post hoc Tukey test for multiple comparisons. A *P* < 0.05 was considered significant. All statistical analyses were done using Origin, Version 2019b. OriginLab Corporation, (Northampton, MA)

## RESULTS

**Figure 4.**
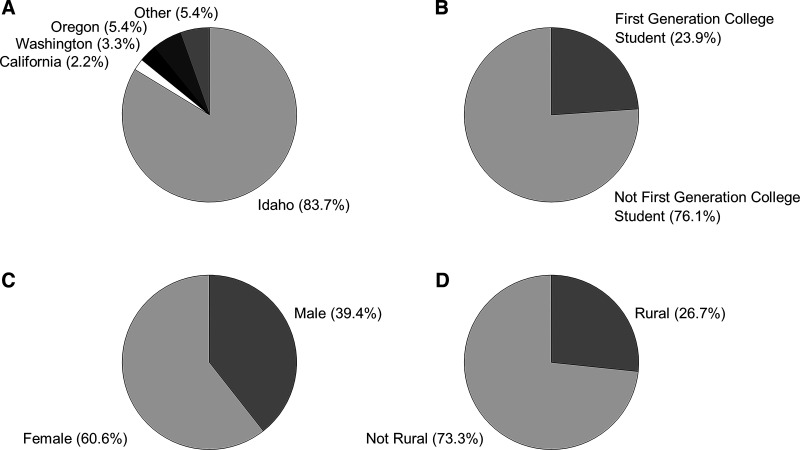
Demographics of the INBRE summer fellowship participants. The pie charts show the percentage of student participants by location of where they graduated from high school (*A*), first-generation college status (*B*), gender (*C*), and rural status (*D*) based on the location of the last high school attended and that location’s eligibility status for Rural Health Grants from the U.S. Health Resources & Services Administration. IDeA, Institutional Development Award; INBRE, IDeA Network of Biomedical Excellence.

Since 2005, a total of 359 students have applied to The College of Idaho INBRE summer fellows program and 103 were selected for participation (acceptance rate of 28.7%). A total of eight faculty (5 biologists and 3 chemists) served as summer mentors during this time. A subset of these students (10) completed two summer fellowships. Figure 4 summarizes the demographic profiles of The College of Idaho INBRE fellows from 2005 to 2019 based on information provided by the student in their application. The majority of fellows, 83.7%, were from Idaho, with a smaller percentage originating from other states. Summer fellows self-identified as either female (60.6%) or male (39.4%) and 23.9% self-identified as being a first-generation college student (defined as neither parent having completed a bachelor’s degree). Students attending high school in a rural area represented 26.7% of the total. Based on internal data from The College of Idaho Registrar’s office, 99% of former INBRE fellows graduated with a bachelor’s degree.

**Figure 5.**
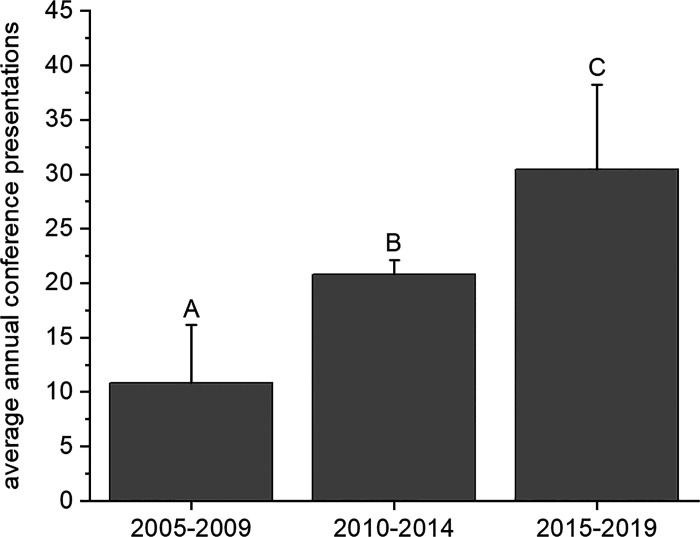
Scientific conference presentations from The College of Idaho undergraduates and faculty who participated in INBRE-funded research from 2005 to 2019. Each bar represents the means ± SD for a 5-yr period (2005–2009, 2010–2014, and 2015–2019). Groups with different letters are significantly different from each other (^A,B,C^*P* < 0.05). IDeA, Institutional Development Award; INBRE, IDeA Network of Biomedical Excellence.

**Figure 6.**
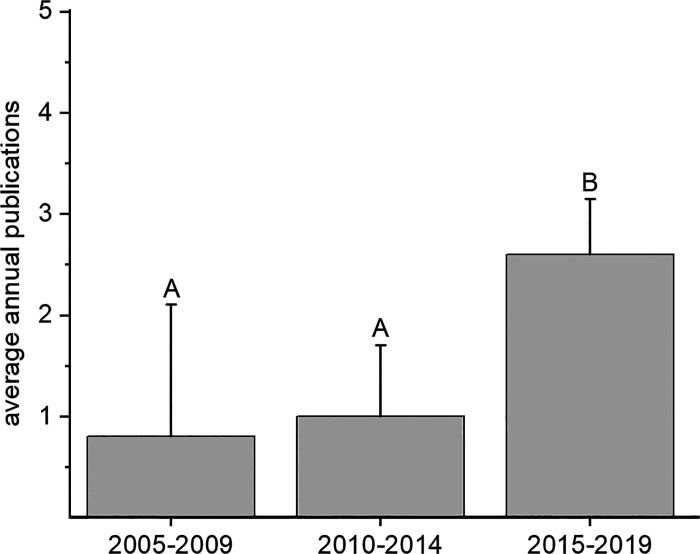
Publications from The College of Idaho undergraduates and faculty who participated in INBRE-funded research from 2005 to 2019. Each bar represents the means ± SD for a 5-yr period (2005–2009, 2010–2014, and 2015–2019). Groups with different letters are significantly different from each other (^A,B^*P* <0.05). IDeA, Institutional Development Award; INBRE, IDeA Network of Biomedical Excellence.

The 10-wk summer research program culminated with each student presenting his or her research at the statewide IDeA INBRE Research Conference. In addition to the annual INBRE conference, students and faculty presented their research at regional (Murdock College Science Undergraduate Research Conference, Idaho Academy of Science and Engineering, etc.) and national scientific conferences (American Chemical Society, Society of Toxicology, Society for Neuroscience, etc.). Figure 5 demonstrates a significant growth in the number of conference presentations by INBRE-funded students and faculty from 2005 to 2019, from ∼10 presentations/yr to 30/yr, a threefold increase. This growth is consistent with the faculty summer mentor productivity expectations of presenting at a conference annually and publishing every other year. Similarly, participation in INBRE resulted in a significant increase (3.3-fold) in the number of peer-reviewed publications by INBRE-funded students and faculty (Fig. 6).

To evaluate the long-term impact, effectiveness, and career outcomes of The College of Idaho INBRE program, former INBRE summer fellows were contacted by email and asked to complete an online survey. A total of 61 of the 103 former fellows completed the survey, a 59% response rate. Former INBRE fellows were asked to give feedback about their experience with the INBRE program. Table 1 summarizes the results from eight Likert scale questions (1 strongly disagree; 2 disagree; 3 neutral, neither agree or disagree; 4 agree; and 5 strongly agree). The survey responses ranged from 4.12 to 4.95 with a mean rating of 4.54. Participants gave the highest mean ratings to the statements “participation in INBRE increased my ability to conduct science” (4.95) and “participation in INBRE enhanced my self-confidence in the laboratory” (4.87). The lowest ratings were given in response to the statements “participation in INBRE enhanced my written communication skills” and “influenced my career decision” (4.12 and 4.16, respectively).

**Table 1.** INBRE summer fellows rating of their research experience

Survey Question: Participation in INBRE	Means ± SD Rating
Influenced my career decision	4.16 ± 0.82
Increased my interest in science	4.68 ± 0.54
Improved my ability to conduct science	4.95 ± 0.22
Increased my competitiveness to be accepted to postgraduate programs	4.58 ± 0.82
Improved my oral communication skills	4.43 ± 0.74
Improved my written communication skills	4.12 ± 0.83
Improved my ability to critique and analyze scientific literature	4.56 ± 0.67
Enhanced my self-confidence in the laboratory	4.87 ± 0.34

Means ± SD were calculated by averaging the responses to each question on the 5-point scale. Participants rated 8 questions on a Likert scale of 1–5 (1: strongly disagree; 2: disagree; 3: neutral, neither agree or disagree; 4: agree; and 5: strongly agree). IDeA, Institutional Development Award; INBRE, IDeA Network of Biomedical Excellence.

In the same anonymous survey, former summer fellows were asked two open-ended questions regarding their College of Idaho INBRE research experience. We received 53 open-ended responses to each of these questions. Table 2 gives representative written responses to the questions “what is the most impactful part(s) of being involved in INBRE-funded research at The College of Idaho” and “is there anything else that you want to share with us as you reflect upon your INBRE-funded research experience?” The responses were categorized into the four major themes of mentoring and relationships, training and technical skills, awareness of scientific opportunities, and postgraduate preparation. Student responses were overwhelmingly positive about their INBRE experience. Written responses supported the Likert scale rating questions with high praise for research training. Although not asked about in the Likert scale rating questions, mentoring was discussed by several former fellows as being the most impactful. One student stated, “The opportunity to do research and work one-on-one with a faculty mentor was a key factor in my decision to pursue a career in science. Even 10 yr later my INBRE fellowship remains on my CV.”

**Table 2.** Representative responses to open-ended survey questions categorized by theme

Mentoring and Relationships	Training and Technical Skills	Awareness of Scientific Opportunities	Postgraduate Preparation
“The opportunity to do research and work one-on-one with a faculty mentor was a key factor in my decision to pursue a career in science. Even 10 years later my INBRE fellowship remains on my CV.”	“Other impactful aspects of the experience included: the importance of attending conferences, disseminating findings, being pushed outside of my comfort zone, and innovation.”	“INBRE is one of the programs that jump-started my career trajectory and opened my eyes to the breadth of research opportunities and paths that are available to young scientists.”	“I started a PhD program immediately after undergrad, and I am 100% sure that I would not have been able to do that without my INBRE-funded research experience.”
“As a first generation, female, minority student, who didn't feel at the time they had the grades to be involved in the program, earning the summer internship and having the ability to continue the research through the academic year was truly a life changing experience.”	“Learning to analyze data and statistics and learning to read journal articles has shaped my ability to practice evidence-based medicine as a PA.”	“Besides doing actual important research, it really opened up a world that seemed out of reach before. I had never really thought about being a scientist before that.”	“Conducting bench research and compiling that work for publication and for oral presentations gave me realistic expectations for graduate school and continuing into academia. The work allowed me to take ownership of my research and made me want to pursue a PhD in Toxicology. I don't think I would have had the confidence to go into a graduate program without my INBRE experience.”
“The professors always knew exactly where to guide me even when I felt completely stuck, lost or confused. This experience allowed me to grow as a person, taking me out of my comfort zone, to areas I could never imagine.”	“Learning to work and make research decisions independently was absolutely the most valuable and transferable skill for graduate school.”	“INBRE was the most valuable educational experience in my undergraduate career. I would not be in a graduate program without this experience or have a fundamental understanding of biomedical research.”	“Ignited my interest in laboratory science and influenced my decision to pursue a PhD in Biochemistry.”

The INBRE summer research fellow quotes are in response to the open-ended questions on their INBRE-funded research experience and are grouped into the 4 major themes of mentoring and relationships, training and technical skills, awareness of scientific opportunities, and postgraduate preparation. IDeA, Institutional Development Award; INBRE, IDeA Network of Biomedical Excellence.

**Figure 7.**
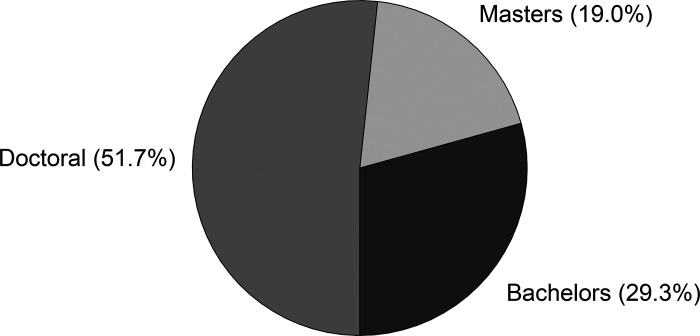
INBRE summer fellows highest degree earned or currently working toward completion. Survey participants were asked to indicate what was the highest degree they had earned or were currently working toward as an enrolled student. IDeA, Institutional Development Award; INBRE, IDeA Network of Biomedical Excellence.

**Figure 8.**
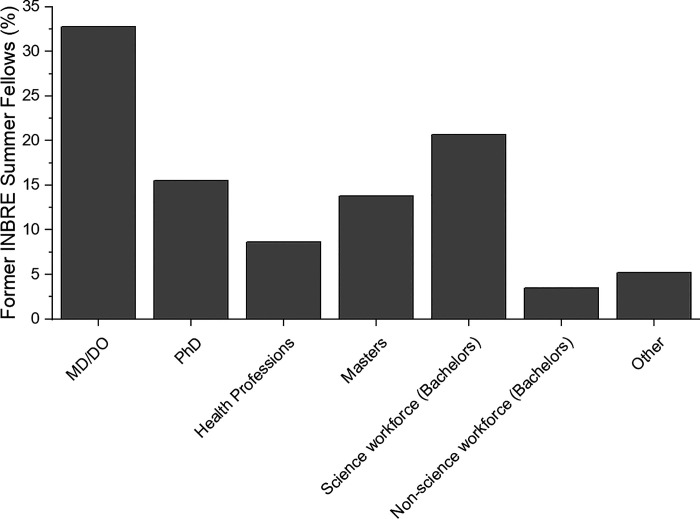
Career choices of INBRE summer fellows. Participants were asked to state their career path based on the options provided. IDeA, Institutional Development Award; INBRE, IDeA Network of Biomedical Excellence.

The third component of the survey was assessing academic and career outcomes of former INBRE summer fellows who have graduated (58 of 61 survey respondents). Figure 7 shows the survey results regarding highest degree earned or currently working toward completion. We found that 51.7% and 19.0% of former INBRE summer fellows have completed or are working toward a doctoral degree and master’s degree, respectively. Figure 8 indicates the career choices of the former INBRE fellows. Among the doctoral-trained individuals, the majority pursued a MD/DO or combined MD/PhD degree (32.8%) or PhD in a science field (15.5%). Two former fellows completed a PharmD (3.4%, included in Fig. 8 with other health professions). We found that 13.8% pursued a master’s degree in scientific disciplines (nonclinically oriented), while 8.6% were employed or in the process of training for a health profession such as physical therapy, pharmacy, or physician assistant (5.2% completed master’s level clinical preparation and 3.4% completed PharmD programs as indicated above). Another anticipated outcome of the biomedical training pipeline is preparing students for bachelor’s level scientific careers. The results indicated that 20.7% entered the science workforce after completing their bachelor’s degree. Collectively, of the survey participants who have graduated from The College of Idaho 91.4% entered biomedical- or science-related professions.

**Figure 9.**
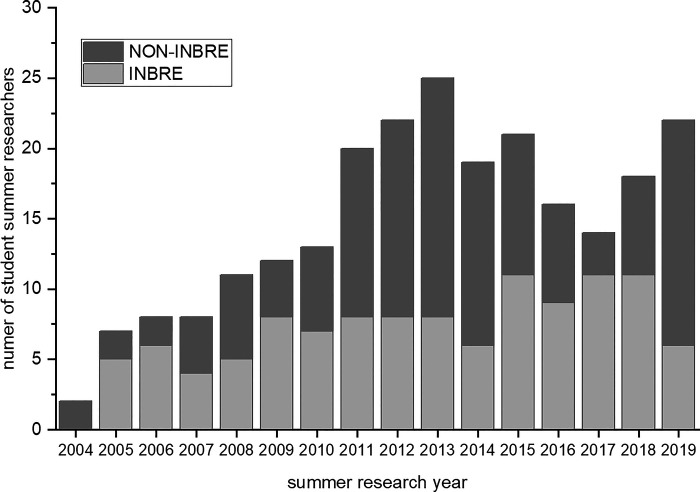
Number of student summer researchers in biology and chemistry from 2004 to 2019. The light gray bar represents INBRE-funded students. The dark gray bar represents NON-INBRE-funded students who were supported by internal college or external federal or nonfederal awards other than INBRE. IDeA, Institutional Development Award; INBRE, IDeA Network of Biomedical Excellence.

The final analysis was to examine the total number of students participating in full-time summer research with a biology or chemistry faculty mentor. Before 2005, The College of Idaho had relatively few faculty and students participating in research. For example, there were only two summer student researchers in 2004. Figure 9 documents the overall growth in summer student researchers (INBRE and non-INBRE funded) from 2004 to 2019 per year. To determine whether the numbers of INBRE and/or non-INBRE student researchers increased significantly over time, we averaged the number of participants in 5-yr blocks and tested for significance. In aggregate, the 5-yr average total number of students (means ± SD) participating in summer research nearly doubled from 9.2 ± 2.2 (2005–2009) to18.2 ± 3.3 (2015–2019). This increase is attributed to growth in both INBRE-funded (5.6 ± 1.5 from 2005 to 2009 to 9.6 ± 2.2 from 2015 to 2019) and non-INBRE-funded summer students (3.6 ± 1.7 from 2005 to 2009 to 8.6 ± 4.4 from 2015 to 2019). All of these increases were statistically significant (Student’s *t* test, *P* < 0.05).

## DISCUSSION

The INBRE summer fellows program greatly impacted The College of Idaho both directly and indirectly. Before 2005, The College of Idaho had relatively few faculty and students actively participating in research. From 2005 to present, the Idaho INBRE program has provided consistent funding for research, training opportunities for students, resources for research laboratory renovations, key new instrumentation, support for core facility usage at other institutions, and networking and professional development opportunities for students and faculty at The College of Idaho. This report focuses on the signature INBRE summer fellows program. The benefits of involvement in this program can be grouped into four major goals, as shown in Fig. 3.

A primary goal was improving student access to research opportunities. There is broad consensus that participation in research as an undergraduate enriches the educational experience in both STEM and non-STEM areas (8, 10–12). Furthermore, the benefits of an undergraduate research experience carry forward with influencing career decisions, increasing competitiveness for acceptance to postgraduate programs, and improving self-confidence as a scientist (11, 13). Several states have reported their outcomes and success of INBRE undergraduate research experiences (14–16).

Since 2005, The College of Idaho provided 103 students with a summer research experience, of which 83.7% were from Idaho. Our data show a remarkably positive impact on these students. Of the students who participated as summer fellows, 102 of 103 graduated (99.0%) as compared with our college-wide graduation rate of 66.5% (the nationwide rate at private colleges and universities is 65.4%). Though this high percentage likely reflects the academic preparation of students who are selected for the fellowship, it does suggest that research opportunities directly correlate with increased graduation rates. In terms of demographics, we found that the students involved in the INBRE summer fellows program were largely similar to The College of Idaho student body at large. The gender of students involved in INBRE were 60.6% female and 39.4% male, and 23.9% were first-generation college students. Lastly, there is a paucity of literature regarding the participation of students in underrepresented groups participating in undergraduate research experiences (17). Thus we examined the percentage of former students that identified as first-generation college students or originating from a rural hometown. We found that a substantial number of INBRE fellows originate from rural communities (26.7%) as Idaho is a rural state. In summary, the INBRE program provides research opportunities to a group of students that is demographically representative of our general student population.

There are clear benefits to the student educational experience by participating in undergraduate research. Our results are similar to a study conducted at PUIs participating in the North Dakota INBRE program. In both studies, summer undergraduate research students gave the highest marks on a Likert scale to increasing self-confidence in the laboratory and increasing ability to conduct science (16). Building self-confidence was also highlighted by students participating in the Kansas INBRE program (18). These results are consistent with undergraduate research programs, which are in many cases 10-wk full-time summer experiences, placing an emphasis on conducting laboratory work (19). Interestingly, the North Dakota INBRE study also reports the lowest rating by students to the statement, “influenced my career decision.” Reasons for this may be because these students are already interested in science when they applied to the program and lean toward pursuing a career in science. This interpretation is supported by written student comments and Likert scale responses that give high praise to INBRE participation increasing their competitiveness for postgraduate programs, suggesting they already were interested in those career paths and that involvement in the INBRE summer fellowship reinforced their commitment to pursue postgraduate education.

A key finding of this study is the value students place upon mentoring and relationships. Mentoring is important in training undergraduate students (20, 21), as also noted by the Arkansas INBRE program (15). About half the students who completed our survey commented on the importance of a faculty mentor in their research experience and in shaping their career trajectory. In a study by Houser et. al (22), undergraduate students completed a survey before and after completing a summer National Science Foundation (NSF) Research Experience for Undergraduate (REU) program. The authors concluded that the most important element of the research experience was the relationship between the faculty mentor and student. This relationship influenced faculty and student research productivity as well as the student’s likelihood to pursue postgraduate education (22).

A second goal of our analysis was an increase in faculty research productivity (Fig. 3). Our findings are consistent with another study that demonstrates enhanced faculty research productivity (publications, presentations, grants) when faculty work with undergraduate researchers (23). We report a significant increase in INBRE-funded conference presentations and publications by faculty and students over the last 15 yr with a particularly large increase in the last 5 yr. This may be due, in part, to sustained faculty involvement in the INBRE renewals. For example, all five faculty members involved in the last year of INBRE were involved each year. In contrast, the first 5 yr of INBRE had only three faculty involved and only one for the entire period. This increase in research productivity, in part, is the result of INBRE investment in faculty time (20–50% effort) and in some cases teaching release during the academic year. Although initially some faculty and administrators expressed reservations regarding an increased focus on research, this dampened over time as students, faculty, and administration saw the benefits of undergraduate research to the educational experience. Being a teaching-centered institution, challenges still remain in balancing heavy teaching loads with mentoring students in research. For example, ongoing challenges include staff support for equipment maintenance and repair, administrative support for teaching release and grantsmanship, and faculty substantial teaching and service commitments during the academic year.

Increase in faculty research productivity included securing external grant funding. Several options have existed for faculty to participate in INBRE-funded research at The College of Idaho, from serving as a Summer Mentor (2.5 mo of summer effort) to Project Investigator (50% effort including academic year teaching release). Productivity obligations correspond to the level of commitment of the faculty investigator, and we observe a direct relationship between required faculty research effort and research productivity. For example, we had three faculty selected to participate at 50% effort who were required to submit an NIH R-type or similar size grant. These faculty were paired with a research-focused faculty member at Boise State University. These collaborations led to two funded NIH R15 research grants and one NSF research grant totaling approximately $500,000. Funding of these grants also coincide with an increased number of non-INBRE-funded students in years 2013 and 2019 (Fig. 9). In addition, the college received a MJ Murdock Charitable Trust research grant in 2019 which contributed to the high number of student researchers that year. Hence an important benefit from participation in the Idaho INBRE program is that The College of Idaho faculty are more competitive in securing external federal or nonfederal awards other than INBRE, which includes an educational grant from Howard Hughes Medical Institute to provide a research-based curriculum for our Introductory Biology course. The more active research environment has also helped attract new tenure-track hires and has led to three MJ Murdock Charitable Trust research start-up grants for INBRE faculty. It is now standard practice to provide a 2-yr research start up package for all faculty in the sciences. Because faculty are securing extramural funds, it has been necessary for The College of Idaho to invest in a Grants Office with a dedicated staff member for pre- and postgrant management. This indicates the transformative nature of INBRE on building research capacity on a PUI campus.

The INBRE program was the catalyst for growth in biomedical research opportunities on our campus. However, such growth could not occur without the necessary supporting infrastructure (Fig. 3). Before INBRE, there was no dedicated research laboratory space in Boone Science Hall. The College embarked on two building remodels (2009 and 2014) that included dedicated research space in the biology and chemistry departments. With the support of the Idaho INBRE Program, the research space now houses new laboratory equipment to support biomedical research, including dedicated space for cell culture, advanced microscopy and digital imaging capacity, and high-performance liquid chromatography. Additionally, INBRE has providing funding for much of our equipment that is found in standard biomedical research facilities, including autoclaves, plate readers, centrifuges, thermal cyclers, etc. These infrastructure investments provide students and faculty the space and equipment necessary to carry out projects related to the state-level theme of cell signaling. Since 2005, ∼442 students per year who are pursuing STEM-related major or minors use instrumentation in teaching and research laboratories. These instruments were, at least in part, funded by the Idaho INBRE program. Furthermore, the INBRE investments in equipment have been leveraged to gain additional instrumentation funding including a recent award from the MJ Murdock Charitable Trust to purchase a new liquid chromatography mass spectrometry with users from multiple science departments. This is one example of researchers on our campus collaborating to advance the undergraduate research experience.

A key aim of the Idaho INBRE program is to establish a climate of statewide collaborations and sharing of resources. This is successfully done through a variety of networking opportunities sponsored by Idaho INBRE (Fig. 3). A hallmark networking event is the annual statewide IDeA INBRE Research Conference. The conference is attended by faculty, postdoctural researchers, undergraduate and graduate students, and administrators from across the state. The conference agenda includes a keynote speaker, professional development workshops, networking events for students and faculty, research presentations, and a poster session that showcases all INBRE summer fellows. Students also value the importance of networking with each other. Several former INBRE fellows commented in the survey on the value of presenting their work and rated their improvement in oral communications high (4.43 Likert scale mean rating). A recent study examined five REU programs and surveyed 88 student participants. A key finding was the value students place on a supportive environment that includes other student researchers who share common goals and interests (9). Besides the benefits to students, the INBRE network has brought benefits to The College of Idaho in a number of other ways. For example, it has enabled The College of Idaho to adopt standard research safety and training protocols, including the creation of chemical use and disposal plans, biosafety training programs, and RCR training. As discussed above, the INBRE network has also facilitated a number of research collaborations, primarily with faculty at Boise State University. Lastly, the network has provided core facilities that greatly expand the equipment available to The College of Idaho faculty. Specific collaborations have involved the use of confocal microscopes, flow cytometers, cryostats and other histology sample preparation materials, plasmid preparation services, and mass spectrometry. These networking opportunities are essential to the Idaho INBRE program, in part, because of our geographic isolation and distance among college and universities in the state.

The four major goals discussed above have created a pipeline for College of Idaho students to health research careers and the scientific workforce (Fig. 3). Students uniformly credit their INBRE experience as the pivotal event that gave them the confidence and skills to move forward in their education and pursue scientifically oriented career goals. Career paths of former summer research fellows resembles those described by other INBRE-funded states. For example, the percentage of students pursuing a PhD from North Dakota PUIs, Kansas, and Oklahoma was reported as 15.3%, 37%, and 11% compared with 16% from The College of Idaho. Notably, we report that 33% of former summer fellows pursued an MD/DO degree which is higher than North Dakota PUIs (7.2%), Kansas (19%), and Oklahoma (11%) (14, 16, 18). The College of Idaho is known for their premedical training and may preferentially attract students who plan to pursue medicine. Collectively, 91.4% of graduates entered biomedical- or science-related professions. A small subset of students was selected for two summer fellowships. We were curious if completing two rather than one summer fellowship would increase their likelihood of pursuing biomedical or science-related professions. Interestingly, we found that those students who did two fellowships (90%) went on to biomedical careers at a similar rate to those who completed only one (91.4%). Hence, we are successfully addressing a key aim of the Idaho INBRE program of enhancing the science and technology knowledge of the Idaho workforce.

A main finding in this study is the role of the INBRE program as a catalyst to significantly increase the total number of student summer researchers since 2005. The reasons for this are threefold. First, INBRE investments have allowed faculty to develop research programs and obtain preliminary data that have been used to secure further extramural funding to support additional non-INBRE students. Second, the increased research capacity of the science departments has strengthened the overall culture of research in the sciences and made The College of Idaho more competitive for institutional grants, including research startup grants from the MJ Murdock Charitable Trust. Third, the INBRE program fostered a supportive climate of cooperation and shared resources across multiple faculty research laboratories, which has had the derivative benefit of increasing both INBRE and non-INBRE faculty research activity. For example, the College secured a 2-yr institutional grant from the MJ Murdock Charitable Trust (2018) to fund undergraduate research in the sciences. This last point is a key step toward creating a sustainable research culture in the sciences, which includes an administrative commitment to establishing a science research endowment.

Besides serving as a catalyst for biomedical research at The College of Idaho, the INBRE program has facilitated a transformative paradigm shift in the culture of undergraduate research campus wide. The visibility of INBRE and other research in the sciences, along with isolated research activity across campus, led to the creation of an annual undergraduate research conference to highlight student research and creative activities across a wide-range of disciplines from humanities, social sciences and natural sciences. The student research conference, now in its 16th year, demonstrates how INBRE has fostered a sustainable culture of research on campus. The College has also established a student research grant program to fund student research and travel to conferences. Lastly, the robust culture of research warranted the creation of a new position, Director of Student Research and Creative Activities, to coordinate these programs across campus.

This study identified many successes of The College of Idaho INBRE summer fellows program. Overall, students were quite positive about their INBRE research experience, although the authors acknowledge the limitations of drawing conclusions based on only postsurvey data. Furthermore, it is important to reflect and continually adjust the program based on assessment and student feedback. In written survey responses, a few students suggested increasing the number of opportunities for student and faculty researchers to network on campus during the summer. Likewise, INBRE faculty had also recognized that the program could be strengthened by more interactions between laboratory groups. In cooperation with the MJ Murdock Charitable Trust Ramp-up grant program, we now have a formalized summer program with weekly sessions for faculty and student that cover topics such as career development, oral and written communication skills, journal clubs, and rotating laboratory research presentations. In the future, we will add a programmatic emphasis on writing skills to address what was rated by students to be an area we could improve upon.

Figure 3 summarizes the goals of The College of Idaho INBRE summer fellows program. This report shows that the program is successfully meeting the goals of the broader NIH IDeA initiative. Consistent with data from other INBRE states, this suggests that summer research programs are a key component of improving biomedical education at primarily undergraduate institutions.

## GRANTS

This publication was made possible by an Institutional Development Award (IDeA) from National Institute of General Medical Sciences Grant P20-GM-103408.

## DISCLOSURES

No conflicts of interest, financial or otherwise, are declared by the authors.

## AUTHOR CONTRIBUTIONS

S.J.H., C.J.H., S.A.M., L.E.L., and R.L.D. conceived and designed research; S.J.H., L.E.L., and R.L.D. analyzed data; S.J.H., C.J.H., S.A.M., and R.L.D. interpreted results of experiments; S.J.H. and R.L.D. prepared figures; S.J.H. and R.L.D. drafted manuscript; S.J.H., C.J.H., S.A.M., and R.L.D. edited and revised manuscript; S.J.H., C.J.H., and R.L.D. approved final version of manuscript.
